# Pose Estimation of Coil Workpieces by Automated Overhead Cranes Using an Improved Point Pair Features Algorithm

**DOI:** 10.3390/s25051462

**Published:** 2025-02-27

**Authors:** Yongbo Zhuang, Jianli Man, Yuchen Jiang, Qingdang Li, Mingyue Zhang

**Affiliations:** 1 College of Automation and Electronic Engineering, Qingdao University of Science and Technology, Qingdao 266061, China; 13665429916@139.com (Y.Z.); 4023041029@mails.qust.edu.cn (J.M.); 15589920882@163.com (Y.J.); 2 College of Sino-German Science and Technology, Qingdao University of Science and Technology, Qingdao 266061, China; lqd@qust.edu.cn

**Keywords:** pose estimation, coil workpieces, point cloud processing, point pair features, generalized ICP

## Abstract

To facilitate the automation of crane operations for grabbing coil stacks in port storage areas, thereby streamlining the processes of warehousing, stacking, and transshipment for enhanced operational efficiency, this paper utilizes algorithms related to 3D point clouds for the pose estimation of coil workpieces. To overcome the limitations of the traditional point pair feature (PPF) algorithm, a novel point cloud registration algorithm is introduced. This algorithm harnesses the advantages of the PPF algorithm in describing local features and integrates it with the Generalized Iterative Closest Point (GICP) algorithm to enhance the robustness and applicability of registration. Finally, comparative experiments demonstrate that the proposed algorithm delivers superior performance. The average pose estimation errors for one, two, and three coils are 1.1%, 1.1%, and 1.2% of the coil size, respectively, with total processing times of 3.6 s, 3.4 s, and 4.7 s, meeting the practical application requirements in terms of accuracy and timing.

## 1. Introduction

With the advancement of industrial levels and innovative technologies, unmanned and intelligent technologies have been further enhanced. This is also the case in the smart port sector, where various types of lifting machinery and transportation devices are indispensable during port cargo handling. While unmanned container transportation has been achieved in some automated terminals, the handling of non-standard cargo (e.g., irregular goods and bulk materials, such as coiled steel, ores, etc.) still relies heavily on manual or semi-automated equipment. Compared to standardized containers, non-standard cargo in ports exhibit significant diversity in categories (e.g., steel pipes, timber, bagged bulk cargo) as well as variations in shape and weight, making standardized grasping extremely challenging. Dynamic operational scenarios—including loosely stacked cargo, suspension swing during hoisting, and human–machine collaborative environments—impose stringent demands on automated systems. Consequently, most ports still depend on experienced operators to control cranes or forklifts.

The primary technical challenges stem from the following. The irregular geometry of non-standard cargo complicates precise boundary detection and grasp point localization using vision algorithms. Customized end-effectors (e.g., electromagnetic suction cups and mechanical grippers) are often required for different cargo types, resulting in high switching costs. Algorithms struggle with real-time responsiveness and robustness under variable lighting, occlusions, and mechanical vibrations. Although a few pilot projects have adopted vision-guided robotic arms for handling non-standard cargo, their stability and efficiency remain suboptimal due to challenges such as shape variability and disordered stacking. 

Therefore, enhancing the automation and intelligence of workshops is crucial. To improve the automation of port coil [1] warehouses, reduce labor costs, alleviate the workload of workers, and decrease the risk factors associated with transporting and lifting large, heavy goods, as well as enhance operational efficiency, an upgrade and transformation toward intelligent and automated port storage systems have been implemented. This transformation involves the entire operational process of the coil warehouse, achieving comprehensive automation and the unmanned management and control of key links, including warehousing, stacking, transshipment, and loading. Consequently, it is essential for the machinery to intelligently recognize the pose of the goods to accurately locate and handle the cargo. This capability of unmanned systems to recognize the position of vehicles and pedestrians ahead and for port lifting machinery to identify the pose of cargo is known as pose estimation.

When acquiring point clouds, different sensors are affected by the hardware of the sensors themselves and environmental factors, resulting in various methods of acquiring point clouds and differing accuracies of the acquired point clouds. For instance, common point cloud sensors such as LiDAR [2], structured light scanners [3], and Time-of-Flight (TOF) cameras [4] are each suited to different application areas.

After acquiring the scene point clouds, the next step is to process the point clouds through a series of operations. Common point cloud processing algorithms include filtering, segmentation, and fitting, which can be implemented using point cloud processing libraries such as PCL [5] or Open3D [6], depending on the programming language or the complexity of the task. Additionally, the CloudCompare software (version 2.13.2) [7] can be used more conveniently for the preprocessing of point clouds. For example, pass-through filtering [8] can be used to remove parts of the scene outside the target point cloud; statistical filtering or radius filtering can be used to remove noise from the point clouds, enhancing the accuracy of subsequent pose estimation; voxel filtering [9] can replace other points within a voxel with the center point of the voxel block, reducing the amount of point cloud data and increasing the speed of subsequent processing. To address different usage scenarios, researchers have improved existing point cloud processing algorithms [10,11], enhancing processing accuracy and better preserving the information of the target point cloud. In the point cloud preprocessing phase of this paper, pass-through filtering [8] combined with CloudCompare was used to filter out ground point cloud information, retaining more characteristic coil-related point clouds and ensuring the accuracy of subsequent pose estimation.

To determine the location of coils [1] in the storage area, facilitating the automated overhead crane’s grabbing process, one of the most common methods in the field of machine vision is to ascertain the position of objects. The pose estimation of the automated overhead crane grabbing the coils, as discussed in this paper, is achieved through point clouds, thus primarily involving three-dimensional pose estimation. When estimating the target pose, it is crucial not only to accurately measure the target’s position coordinates in three-dimensional space (i.e., positions on the x, y, and z axes) but also to consider the possibility of the target rotating around the x, y, and z axes. Hence, the task of pose estimation actually includes the simultaneous measurement of both translational components in three-dimensional space and rotational components, requiring the consideration of both the object’s translation and rotation.

As is known to all, feature correspondence [12], template matching [13], voting [14], and deep learning [15,16] are four common methods of three-dimensional pose estimation. These methods can be classified into two categories: one is traditional methods and the other is deep learning methods. In recent years, deep learning-based methods have significantly advanced in terms of accuracy and robustness, particularly in complex and dynamic environments. Regarding the application of deep learning in pose estimation, for example, a more reliable framework for 3D pose estimation is presented in [17], while deep learning has also demonstrated strong performance in UAV-based small object tracking and occlusion handling [18,19,20]. These advancements highlight the potential of deep learning in challenging pose estimation tasks. Despite these improvements, the application of deep learning-based methods in our specific scenario presents several challenges. The storage area under consideration features neatly arranged coils with minimal shape variations, which reduces the need for complex feature extraction. Furthermore, deep learning approaches typically require large-scale annotated datasets and extensive computational resources for training and deployment. In practical engineering applications, this translates to higher costs and longer implementation periods.

Traditional pose estimation methods, such as the PnP [21], Linemod [22], and PPF algorithms [23,24], are commonly utilized, and many researchers have made improvements to these algorithms. Among them, the PPF (point pair feature) algorithm is recognized for its high accuracy and robustness and is acclaimed as one of the finest non-deep learning pose estimation methods. Given the unique challenges in the environment of coil workpieces, such as significant shape variations, complex backgrounds, and high real-time requirements, this paper opts for the improved Point-Pair Features (PPF) algorithm rather than template matching or deep-learning methods. During the production, transportation, and storage of wire coils, shape changes may occur, such as deformation and distortion. The template-matching algorithm depends on predefined templates and is quite sensitive to changes in the appearance of the target object, occlusion, etc. For wire coils with significant shape variations, it is difficult to find a universal template for accurate matching. Therefore, it is hard to obtain stable and accurate pose-estimation results using template matching. However, the PPF algorithm, based on point-cloud data, can better adapt to such shape changes by calculating the geometric features between point pairs, making them more suitable for the complex production environment of wire coils.

The environment of coil workpieces usually has a complex background with various interfering factors. Although deep-learning methods can handle complex backgrounds to some extent, they rely on a large amount of annotated data for training, and the training and deployment of models require high computational resources. In the task of wire-coil pose estimation, obtaining a large amount of accurately annotated wire-coil point-cloud data is costly. Moreover, problems such as unbalanced sample distribution may exist in the actual production environment, which can easily limit the generalization ability of deep-learning models. The PPF algorithm, through its local matching and voting mechanisms, does not require a large amount of annotated data and can accurately identify the pose of wire coils in a complex background.

In industrial production, the pose estimation of wire coils usually needs to be completed in real time to adjust equipment or operations promptly. Although the deep-learning-based template-matching algorithm can reduce the number of model parameters through lightweight design, it still has problems such as high video-memory consumption and slow running speed. The fast-voting mechanism and sparse-sampling strategy of the PPF algorithm enable it to complete pose estimation in a short time, which is crucial for industrial scenarios with high real-time requirements. Therefore, this paper employs PPF to determine the pose of coils in the storage area.

Although PPF (point pair feature) performs exceptionally well in coarse registration, its inherent reliance on discrete sampling and voting mechanisms necessitates the use of fine registration to ultimately obtain accurate workpiece poses. The ICP (Iterative Closest Point), a classic fine registration algorithm, is suitable for small-scale, low-noise, and rigid registration scenarios but exhibits limitations when applied to large-scale coil point cloud registration [25].

The Generalized Iterative Closest Point (GICP) algorithm, a significant variant of the ICP, has gained widespread attention and application in point cloud registration. As proposed by Alex Segal et al., GICP addresses the limitations of the standard ICP in handling large-scale point clouds and complex environments [26]. Building upon the standard ICP framework, GICP introduces probabilistic surface characteristics into its formulation. Its core innovation lies in replacing the Euclidean distance metric used in traditional ICP with the Mahalanobis distance, computed using covariance matrices derived from surface normals. By adopting this approach, the GICP algorithm demonstrates superior capability in handling anisotropic noise, making it particularly suitable for point cloud registration in large-scale, noisy/outlier-prone, non-rigid, or dynamic scenarios [27,28,29].

Therefore, this paper bases its study on the PPF algorithm proposed by Drost [19], employing the traditional PPF method for experimentation. It was observed that the traditional PPF algorithm does not perform well in pose estimation for large workpieces like coils using point cloud data. Hence, this paper addresses such issues by introducing the FPFH algorithm [30] to find features and using these features for a coarse registration combining PPF and RANSAC [31], followed by fine registration using the GICP [28] method. These algorithms are applied together in instances of large point clouds like those of coils in the storage area, thus enhancing the accuracy of pose estimation for large coil point clouds.

To enhance coil pose estimation, this study integrates the point pair feature (PPF) algorithm with a Fast Point Feature Histogram (FPFH) [21], Random Sample Consensus (RANSAC) [31], and Generalized Iterative Closest Point (GICP) [28], leveraging their complementary strengths. The PPF captures geometric relationships between point pairs, providing a robust initial alignment. The FPFH enhances local feature descriptions, improving the reliability of correspondence matching. RANSAC eliminates mismatches, ensuring a more accurate initial pose estimation. Finally, GICP refines the pose by minimizing point-to-plane distances, improving both accuracy and stability.

The combination of PPF and GICP is particularly effective and well suited to this application. The PPF establishes a reliable coarse alignment by leveraging geometric invariants, but its accuracy may be affected by noise and partial occlusions. GICP compensates for these limitations by performing fine-grained point-to-plane optimization, significantly improving pose refinement. This hybrid approach enhances robustness in structured storage environments while achieving high precision with lower computational costs compared to purely learning-based methods.

In summary, the main research of this paper is as follows: Section 1 provides a general overview of the current state of coil grabbing and pose estimation in the storage area. In industrial automation, especially when dealing with the pose estimation tasks of coil workpieces in warehouses, the PPF algorithm demonstrates excellent robustness due to its ability to effectively handle variations in surface textures and shapes of the workpieces, as well as its outstanding environmental adaptability. This has made the PPF algorithm the preferred technology for locating and grabbing coil workpieces in complex and variable industrial environments. Therefore, this paper applies these pose estimation algorithms to the pose estimation of coil workpieces in port storage areas. Section 2 briefly introduces the principles of several key algorithms related to the main steps used in this paper, mainly focusing on point cloud acquisition, preprocessing, and the PPF algorithm. Section 3 mainly introduces the improvements and work of this paper, addressing the shortcomings of the traditional PPF algorithm when dealing with large workpieces like coils and its insufficient performance in point pair finding during coil point cloud pose estimation.

In addition, the data that support the findings of this study are available from Port Company, but restrictions apply to the availability of these data, which were used under license for the current study and so are not publicly available. Data are, however, available from the authors upon reasonable request and with the permission of Port Company.

## 2. Algorithm Principles of This Paper

### 2.1. Point Cloud Acquisition and Preprocessing

In the field of 3D computer vision, the acquisition of point cloud data is the first crucial step. Common devices for acquiring point clouds include laser scanners [32], structured light scanners [3], and RGB-D cameras [33]. These devices operate on principles such as Time-of-Flight (ToF), phase shift, or triangulation, each offering unique advantages and limitations under different environmental and lighting conditions. For example, laser scanners excel in long-distance measurements and high precision but are relatively costly; structured light scanners perform better in capturing surface details of objects but are significantly affected by ambient lighting, and their effectiveness diminishes as the distance between the target and the camera increases. Therefore, for the acquisition of point clouds in the large warehouse environments of ports as described, using a laser scanner is more suitable.

Laser scanners are divided into line and planar types. Line laser scanners emit a linear beam to scan an object’s surface and calculate its 3D coordinates based on the deformation of the laser line, generating point cloud data. Planar laser scanners project a laser plane onto an object’s surface, covering a larger area and capturing complete point clouds without movement. In large warehouse spaces, neither type can capture complete point clouds at once, but line laser scanners, with better cost efficiency and scanning precision, are more suitable for this application.

After acquiring point cloud data, preprocessing is an essential step. The primary purpose of preprocessing is to extract the main target point cloud data, eliminate messy noise or unrelated point cloud data, lay the groundwork for subsequent operations, and reduce future computation time. In the data preparation phase, pass-through filtering can be used to set spatial coordinate thresholds for the extensive cropping of point cloud data; in addition, point cloud processing software like CloudCompare can also be utilized for further processing. However, point cloud processing is not always necessary for every single dataset; for point clouds with fewer points, virtually no noise or background, and those that do not affect subsequent processes, preprocessing may not be required.

### 2.2. Coil Point Cloud Preprocessing

As described in Section 2.1, after acquiring coil point cloud data through sensors, the next phase is data preparation. This stage is crucial in the entire process of point cloud processing and pose estimation, as it directly influences the runtime and accuracy of subsequent pose estimation tasks. Therefore, this paper utilizes several common preprocessing algorithms from Open3D [6] for the data preparation phase of coil point cloud pose estimation. The main techniques applied are pass-through filtering, radius filtering, and voxel filtering in the preprocessing of coil point clouds, ensuring the quality of point cloud data and the accuracy of pose estimation.

Pass-through filtering [8] is a range-based filtering technique used to crop point cloud data. It allows for the removal of environmental point clouds not belonging to the target and can also segment regularly repeated target point clouds. In the data preparation phase, pass-through filtering is one of the quickest and most convenient algorithms in traditional methods. It works by setting a well-defined range along coordinate axes, including setting thresholds on the x, y, and z axes to filter out point clouds that are either below or above these thresholds, thereby retaining only the point clouds located at specific heights. This method effectively isolates the point clouds of stacked coil workpieces. For instance, when using the *z*-axis, the relevant formula is as follows (1):

(1) $$P_{filtered}=\{p\in P:z_{\min}\leq p_{z}\leq z_{\max}\}$$

In this process, P represents the original point cloud and $P_{filtered}$ is the point cloud after filtering. $p_{z}$ is the Z-coordinate of each point in the point cloud, while $z_{min}$ and $z_{max}$ are the preset height limits. In the processing of coil point clouds, pass-through filtering can be used to extensively remove non-noise-like point clouds such as ground point clouds and other background scene point clouds. It can also accurately segment different groups of coil point clouds based on the actual grabbing scenarios in port storage areas.

After completing the pass-through filtering [8], radius filtering [11] is applied for noise reduction. Radius filtering works similarly to drawing a circle around each point in the point cloud. It involves iterating over each point, considering the number of neighbor points within a certain radius, and setting a threshold for the minimum number of neighbor points. Points with neighbors fewer than this threshold are filtered out as isolated points. This step is crucial for removing noise points, as these can significantly affect the accuracy of pose estimation. This process is represented by Formula (2):

(2) $$P^{\prime }=\{p\in P_{filtered}:|N(p,r)|\geq \tau \}$$

As shown by the formula, N|(p,r)| denotes the set of neighboring points around point p within radius r, where τ is the threshold for the number of neighboring points and $P^{\prime }$ is the point cloud after filtering. In the context of coil workpieces, since point cloud sensors in the storage area mainly scan the top cylindrical surface of the coils, noise points may appear on other surfaces of the coils. Therefore, using radius filtering helps remove noise points from around the coil workpieces and on their surfaces, especially on the top and bottom circular faces. This prevents noise points from being mistakenly treated as feature points in subsequent pose estimations, which can lead to significant errors or cause the algorithm to fail to produce accurate results.

After completing noise reduction with pass-through filtering [8] and radius filtering [11], the point cloud data are further simplified using voxel filtering [34], depending on the size of the point cloud data and the time requirements of the algorithms used. Voxel filtering [34] involves setting a spatial threshold to divide the space into a regular grid (voxels) and, within each voxel, replacing the points with a centroid or the average point of all the points within that voxel. This process reduces the data volume while maintaining the structural characteristics of the point cloud and increasing processing speed. The expression for voxel filtering is typically represented as shown in Formula (3):

(3) $$P^{\prime \prime }=U_{v\in V}centroid(v)$$

**Figure 1:**
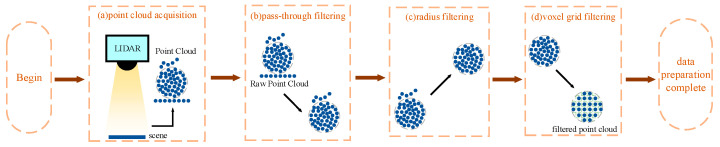
Flowchart of point cloud data acquisition and preprocessing stages.

Here, V represents the set of voxels, and centroid(v) is the geometric center of all points within voxel v. Therefore, in cases involving large volumes of point clouds such as coils, voxel filtering can be used to appropriately reduce the amount of coil point cloud data to shorten the time required for subsequent pose estimation. However, the spatial threshold should not be set too high, as this could lead to a reduction in the number of point clouds after voxel filtering, making it difficult to find suitable feature points for matching. Overall, the process for preparing point cloud data is illustrated in Figure 1 below. This method of acquisition and preprocessing not only ensures data accuracy but also significantly enhances the efficiency and precision of subsequent pose estimation.

### 2.3. Point Pair Feature (PPF) Algorithm Principles

The point pair feature (PPF) algorithm is a technique specifically designed for 3D object recognition and positioning, which works by analyzing the geometric relationships between pairs of points in point cloud data. The PPF algorithm proposed in Drost’s paper [19] is acclaimed to be one of the finest of the non-deep learning three-dimensional pose estimation algorithms. Nowadays, the PPF algorithm is widely applied in the field of 3D vision and is especially significant in object recognition and pose estimation, and many researchers have made specific improvements to the traditional PPF method. For example, Yifan Chen [35] optimized the pre-noise reduction phase of the PPF algorithm and addressed the issue of false positives after the completion of the PPF algorithm in this paper. This paper follows the PPF approach proposed by Drost [19], dividing the algorithm into two phases: offline global training and online local matching.

**Figure 2:**
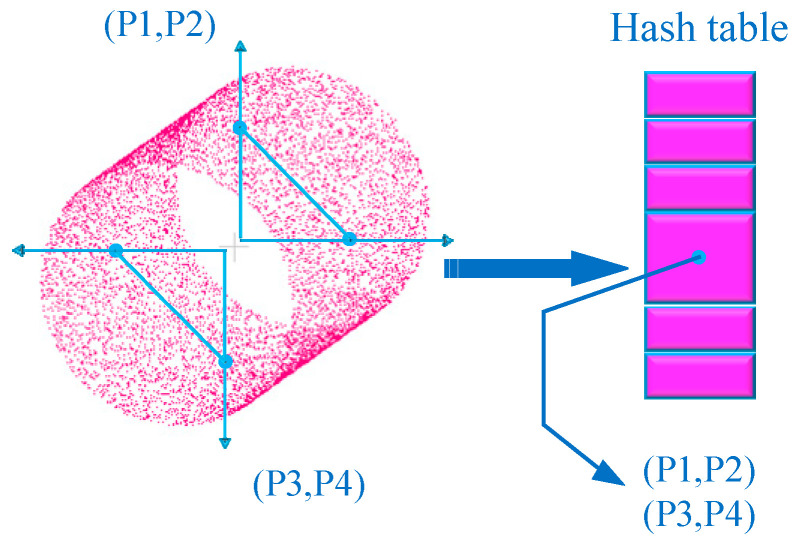
PPF algorithm offline training diagram.

In the offline global training phase, the algorithm first calculates a four-dimensional feature vector for each pair of points in the model point cloud. This vector includes the distance between the two points, the angle between their normal vectors, and the angles between each point’s normal vector and the line connecting the two points. These feature vectors not only describe the local geometric structure of the point cloud but also exhibit invariance to the object’s rotation and translation. Subsequently, these vectors are stored on a hash table, as illustrated in Figure 2.

Using a hash table can reduce the amount of data required for subsequent processes, such as matching, by allowing direct search and extraction of matching point pair features. This avoids the cumbersome process of sequentially eliminating incorrect matching pairs, significantly improving efficiency. In the subsequent online matching phase, for any reference point in the scene point cloud, if the reference point is on the detection target, it is certain there will be a corresponding point from the model. By aligning these two points and their normals and rotating around the normal of the scene reference point by a certain angle, the model can be aligned with the scene. For each model point pair, the rotation angle α can be calculated using the following formula, as shown in Equation (4), where $S_{i}$ and $P_{i}$ are points in the point cloud, and T and R represent the translation and rotation matrices.

(4) $$S_{i}=T_{s\to g}^{-1}R_{x}(\alpha )T_{m\to g}P_{i}$$

Taking a set of point pair features as an example, relationships are established between point pairs through their features, and these relationships are then cast into a Hough space for voting. A threshold is set, and through continuous matching and filtering, the coordinates that do not exceed this threshold and receive the highest votes are retained as the local coordinates. The final output is the pose estimated by the PPF algorithm. Generally, the optimal local coordinates correspond to the true pose of the target object in the scene. However, for stability considerations, further optimization of the target pose is necessary using the Iterative Closest Point (ICP) method. This involves refining the alignment between the target pose point cloud and the scene point cloud.

## 3. Improved PPF Algorithm and Its Application in Storage Areas

### 3.1. Application of the PPF Algorithm to Coil Workpieces in Storage Areas

**Figure 3:**
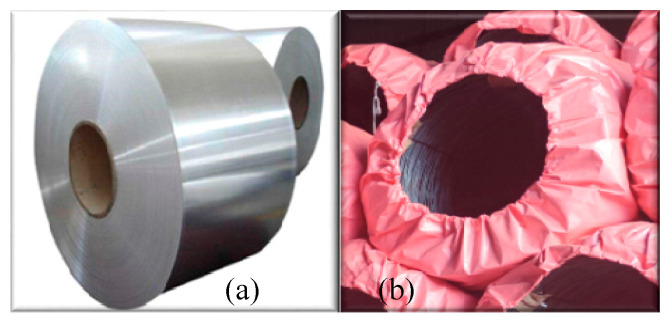
Comparison of appearance between steel rolls and coil workpieces: (**a**) steel roll and (**b**) coil workpiece.

In industrial automation, particularly in tasks such as the pose estimation and grabbing of coil workpieces in warehouses, as discussed in this paper, the main challenges arise from the unique shapes of workpieces and environmental factors, such as coils and steel rolls. As shown in Figure 3, unlike the steel coils discussed in the articles by Lim T G [36] and Liu et al. [37], which have relatively smooth surfaces, where each coil is supported on a fixed saddle without stacking, the surface of coil workpieces in the actual storage areas is not as smooth. Additionally, unlike other smooth or regularly shaped workpieces that can be identified and located through simple reflective features and edge detection, coil workpieces require a more complex processing sequence. This complexity is due to the texture that forms on the surface of the workpieces after the steel wire is coiled, which is not smooth, and their shapes may undergo slight deformations due to stacking during the production stages and transportation methods. Therefore, the surface texture and irregular deformations of coil workpieces may lead to errors in feature matching and model alignment in traditional algorithms.

Through a series of comparative experiments conducted in an actual warehouse environment, the PPF [19] pose estimation algorithm proves to be more suitable than other algorithms due to its high robustness and adaptability. As also indicated in Section 2, the PPF algorithm estimates the pose of objects by calculating and matching the relative geometric relationships between point pairs. This method has a high tolerance for subtle surface details and shape variations in objects. Therefore, it can adapt to these variations in coil workpieces, accurately recognizing and locating them by analyzing geometric features and effectively handling the complex textures and potential minor shape irregularities of coil workpieces.

Additionally, challenges such as variations in lighting, occlusions, and noise within industrial environments like warehouses pose challenges to vision systems. The PPF [19] algorithm’s outstanding robustness is another advantage, as it can mitigate these negative impacts, ensuring stable performance in the positioning tasks of coil workpieces even under adverse conditions. Therefore, after conducting pose estimation experiments with several commonly used traditional algorithms, the PPF algorithm, with its strong stability and adaptability, was ultimately chosen as the ideal basis for the pose estimation of coil workpieces in this paper.

### 3.2. The Improved PPF Algorithm Presented in This Paper

As stated in Section 3.1, the traditional point pair feature (PPF) [19] algorithm was employed for pose estimation experiments on coil workpieces. However, due to the coils’ large volume and dense point cloud data, the traditional PPF algorithm could not achieve satisfactory results in practical registration. The primary issue was identified after using PPF for coarse registration, where the subsequent fine registration using the Iterative Closest Point (ICP) failed to properly align the coil model point cloud with the scene point cloud. This resulted in a significant discrepancy between the ideal and actual output poses, impacting the effectiveness of subsequent crane operations. To enhance the pose estimation accuracy, this paper prioritized the use of the Fast Point Feature Histogram (FPFH) [21] algorithm to calculate point cloud features, followed by a combination of the PPF algorithm, RANSAC [31] for coarse registration, and Generalized ICP (GICP) [28] for fine registration. Additionally, to manage the complexity of the overall pose estimation algorithm, multi-threaded processing was implemented to accelerate the computations, ensuring high precision while minimizing time consumption.

The Fast Point Feature Histogram (FPFH) [21] algorithm is an efficient local feature descriptor that operates on the principle of calculating local histograms around each point in the point cloud to describe the surface characteristics of the point. This is primarily achieved by establishing a KD tree to find the nearest neighbor of each point in the point cloud, then calculating the angles between each point alongside the projection angles of the vector between any two points on the plane of one of those points, and the projection angles of the vector on the tangent plane of one of the points. After these calculations, the influence of neighboring point pairs is further incorporated. Compared to other feature extraction methods, the FPFH provides better robustness and higher feature recognition accuracy in complex environments, making it particularly suitable for use in point clouds with rich details or irregular shapes. For large-scale point cloud data like coil workpieces, the FPFH offers faster processing speeds and accuracy. Therefore, in this paper’s algorithm, the FPFH is used to describe the features of the coil point clouds, providing support for subsequent point cloud registration.

The combination of the point pair feature (PPF) algorithm with RANSAC for coarse registration [31] addresses the issue of insufficient accuracy in pose estimation for large-scale coil workpieces using traditional PPF methods. The RANSAC algorithm, well known and integrated into the Open3D point cloud processing library, is primarily used for shape fitting [38] and implementing coarse registration. By utilizing FPFH’s features to identify correspondences in RANSAC, the algorithm leverages its capabilities for the rough registration of point clouds. This method ensures robust estimation of model parameters even when the data contains a large number of outliers and filters out reliable correspondences from a multitude of potential matches. This provides a more accurate initial transformation matrix for fine registration.

The Iterative Closest Point (ICP) [39] algorithm is one of the classic methods for point cloud registration, which finds the optimal transformation matrix by minimizing the distance error between the point clouds. Although the ICP algorithm has shown good registration results in various application scenarios, it is highly sensitive to the initial pose estimation and can easily fall into local optima. To overcome the limitations of the ICP algorithm, the Generalized ICP (GICP) algorithm was developed. The GICP is an advanced point cloud registration technique that integrates features of both the traditional ICP and point-to-plane ICP algorithms within a probabilistic framework. This algorithm not only considers the positional information of points, focusing on the spatial matching between point clouds but also takes into account the local structure of the point clouds, such as the shape and orientation, thereby enhancing the accuracy and robustness of the registration, as shown in Equation (5) as follows:

(5) $$\begin{array}{c} E(R,t)=\sum_{i}{\left(Rp_{i}+t-q_{i}\right)}^{T}{\left(\sum_{I}\;\right)}^{-1}(Rp_{i}+t-q_{i})\\ \sum_{i}=C_{p}+RC_{q}R^{T} \end{array}$$

The formula above incorporates a cost function that factors in both distances between points and their local geometric structures. Here, $p_{i}$ and $q_{i}$ are the nearest points in the target point cloud Q found from the source point cloud P. Local covariance matrices $C_{p}$ and $C_{q}$ are constructed around these points, with the subsequent formulae involving a covariance matrix R for the rotation matrix and t for the translation vector. Typically, the objective is to iteratively use gradient descent to find the rotation and translation parameters that minimize the cost function. After generating an initial pose matrix using the PPF combined with RANSAC [31] for a coarse estimate of the point cloud pose, the GICP [28] algorithm is applied to further refine the alignment. This approach, which uses the GICP instead of the traditional ICP [40] algorithm, significantly improves the precision of the final registration. The pseudocode for the improved PPF algorithm described in this paper, incorporating FPFH, RANSAC, and GICP for fine registration, is structured accordingly. See Algorithm 1.
**Algorithm 1:** Improved PPF Algorithm(1) Calculate FPFH FeaturesF_S ← Calculate FPFH(S’)F_T ← Calculate FPFH(T’)(2) RANSAC Coarse RegistrationR ← RANSAC(F_S, F_T)S_aligned ← Apply Transformation (R, S’)(3) GICP Fine RegistrationG ← GICP(S_aligned, T’)S_final ← Apply Transformation (G, S_aligned)

Let $S^{\prime }$ denote the source point cloud and $T^{\prime }$ denote the target point cloud used for registration. F_S and F_T are the Fast Point Feature Histogram (FPFH) features of $S^{\prime }$ and $T^{\prime }$, respectively, computed via the calculate FPFH function. These FPFH features depict the local geometric characteristics of the point clouds, serving as the data foundation for subsequent matching and pose estimation.

The transformation matrix R is derived by processing F_S and F_T with the Random Sample Consensus (RANSAC) algorithm, and is then applied to $S^{\prime }$ for coarse registration, resulting in the coarsely registered source point cloud S_aligned.

Subsequently, the Generalized Iterative Closest Point (GICP) algorithm refines the registration between S_aligned and $T^{\prime }$, yielding the transformation matrix G. Applying G to S_aligned gives S_final, the accurately registered source point cloud, which represents the optimized pose-estimation outcome of the improved PPF algorithm.

**Figure 4:**
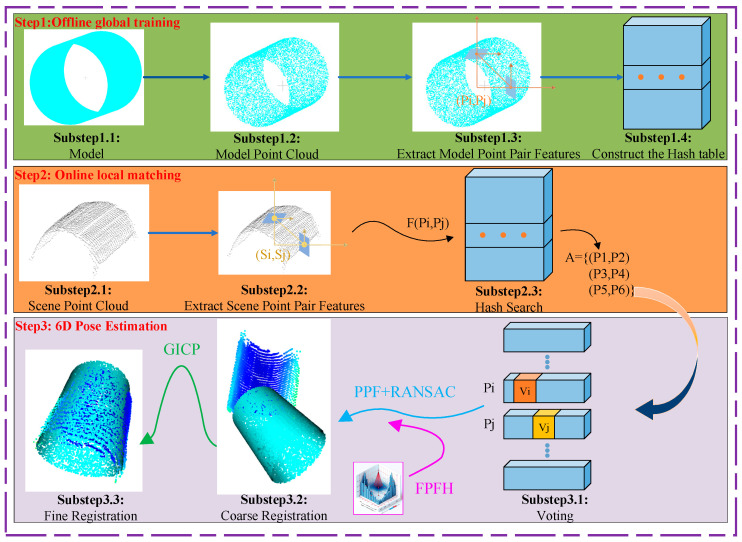
Algorithm flow described in this paper.

The overall algorithm flow for the point cloud pose estimation of coil workpieces in the storage area, as implemented in this paper, is shown in Figure 4. This section is divided into three parts: offline global training, online local matching, and 6D pose estimation. Offline global training includes four components: (1) object modeling to create a 3D representation of the target object; (2) model point cloud construction to generate a sampled point cloud of the object model; (3) model point pair feature extraction to compute point pair features (PPFs) for all pairs in the model point cloud; and (4) hash table construction: discretize PPFs and build a hash table for efficient feature indexing. Online local matching includes three steps: scene point cloud acquisition: (1) capture or generate the scene point cloud; scene point pair feature extraction: (2) compute PPFs for sampled point pairs in the scene; and (3) hash search: for each scene point pair feature, search for similar model point pairs in the hash table (utilizing the hash table built in Substep 1.4). 6D pose estimation consists of three stages: (1) voting mechanism: vote for candidate poses by aggregating transformations from matched point pairs (obtained in Substep 2.3); (2) pose clustering and coarse filtering: cluster similar candidate poses and remove redundancies; (3) ICP refinement: use the top candidate poses from PPFs as initial transformations for the ICP. Then, the pose alignment is iteratively optimized. The pose with the minimal error is selected as the final estimation result.

### 3.3. Establishment of the Scoring System

To more accurately and intuitively reflect the effectiveness of the algorithm presented in this paper, a robust scoring mechanism is introduced alongside the visualization of the pose estimation results. While traditionally, the Root Mean Square Error (RMSE) [41] has been used to assess registration accuracy, it often excludes erroneously registered points by limiting the nearest point distance threshold, resulting in artificially low RMSE values that may not truly reflect the actual quality of registration. To overcome this limitation, the sum of squared distances of corresponding points in the registered point clouds is used as an evaluation metric. This approach, unaffected by the exclusion of erroneous points, considers all paired point distances, providing a more comprehensive assessment of registration quality. A smaller sum indicates better registration accuracy and a larger sum suggests significant errors. The evaluation metric, FitnessScore, is calculated with the following Formula (6):

(6) $$FitnessScore=\sqrt{\sum_{i}{\left(p_{i}-q_{i}\right)}^{2}}$$

In this formula, $p_{i}$ and $q_{i}$ represent the corresponding points in the model point cloud and the scene point cloud after registration, respectively, and the units of the calculated result are the same as those of the input data. Therefore, using this more rational scoring mechanism, the pose estimation results for coil workpieces are quantified, making them easier to verify and apply in practical fields.

## 4. Automated Overhead Crane Grabbing Experiment in the Storage Area

### 4.1. Coil Workpiece Data

**Figure 5:**
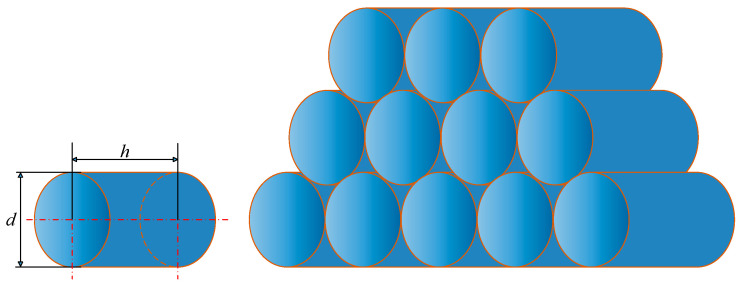
Diagram of coil simulated as a cylinder and coil stacking.

Coil workpieces [1] are goods consisting of steel wire wound into coils, typically enveloped in a protective casing; thus, they can be likened to a cylindrical body, as shown in Figure 5. Here, d represents the diameter of the coil and h denotes the height or thickness of the coil.

Due to variations in the number of wire wraps and the diameter of the initial wrap during production, coil workpieces from the same batch may exhibit some dimensional discrepancies. However, these discrepancies are generally negligible in practical engineering. For this reason, in this paper, the height and diameter of coil workpieces from the same batch and stack are measured, and the calculated averages are used as standard values for the subsequent construction of model point clouds. After measurements in the storage area, the geometric parameters for a single coil are provided, as shown in Table 1 below.

**Figure 6:**
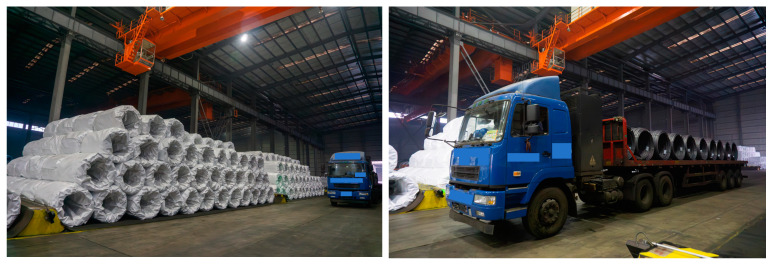
Diagram of coil placement on warehouse ground and truck saddles.

To save warehouse space and enhance transportation efficiency, coils are often stacked in a pyramid shape, as shown in Figure 6 below. Typically, a standard stack on the ground consists of three layers of coils, while truck platforms, depending on the shipment size, may carry single or double layers of coils. To facilitate stacking and prevent rolling in the warehouse, a pair of saddles equipped with concave surfaces is used on both the truck platforms and the ground. When the overhead crane places or grabs a coil, the cylindrical surface of the coil presses tightly against the saddle, ensuring that the coil remains securely fixed on the ground or truck platform.

**Figure 7:**
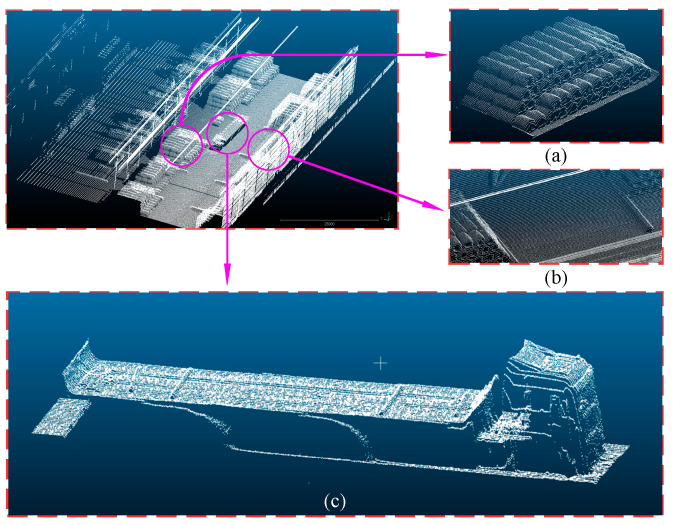
Warehouse scene point cloud diagram: (**a**) ground stacking; (**b**) bare ground point cloud; and (**c**) truck point cloud.

After scanning the entire storage area of coil workpieces with LiDAR (Light Detection and Ranging), a complete point cloud of the warehouse scene was obtained. This included scenes of stacked coils on the ground and on trucks, as shown in Figure 7 below. The left image shows the entire scene point cloud of the storage area. We used CloudCompare to segment the scene point cloud, and the right image (a) displays ground point cloud stacks, including both the bare ground and areas where the coils are stacked. Image (b) shows the point cloud stacks on the truck within the scene, and image (c) depicts the point cloud of the truck in the scene.

**Figure 8:**
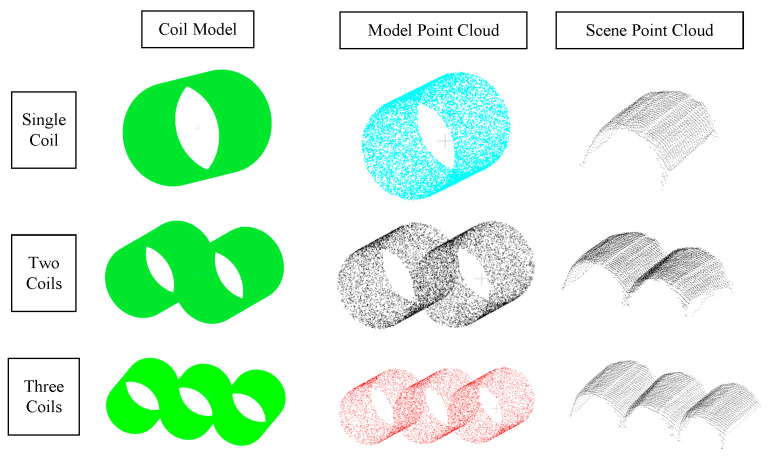
Coil point cloud instance data preparation.

The crane is equipped with a large electromagnetic fixture, which consists of three rectangular electromagnets arranged in parallel. The shape of the electromagnets is designed to perfectly fit the upper cylindrical surface of the coils, with dimensions that closely match the cylindrical surface. As a result, when the fixture is electrified, it can stably lift coils positioned at the same height above the ground in a single layer. In actual overhead crane operations, it is common to grab three coils at a time. However, when the number of coils in a layer is not divisible by three, the remaining coils will be fewer than three, and the fixture may end up lifting only one or two coils. Therefore, in this paper, after general point cloud segmentation and noise reduction preprocessing, pose estimation was specifically conducted for cases with three coils, two coils, and one coil. The results of this data preparation process are illustrated in Figure 8.

In actual overhead crane operations, it is common to grab three coils at a time. However, due to the pyramidal stacking shape of the coils, the number of coils in a single row per layer varies and sometimes is not divisible by three, leading to situations where the crane may need to pick up just one or two coils. To more closely mirror real-world grabbing scenarios, during the data preparation stage, point clouds for individual coils, pairs of coils, and sets of three coils were segmented from the scene point cloud. Using the coil shape and size data described earlier, models of the coils were created and then converted into point cloud data to serve as model point clouds. This preparation facilitated subsequent point cloud preprocessing and pose estimation. The results of this data preparation process are illustrated in Figure 8.

### 4.2. Storage Area Equipment and Operational Methods

**Figure 9:**
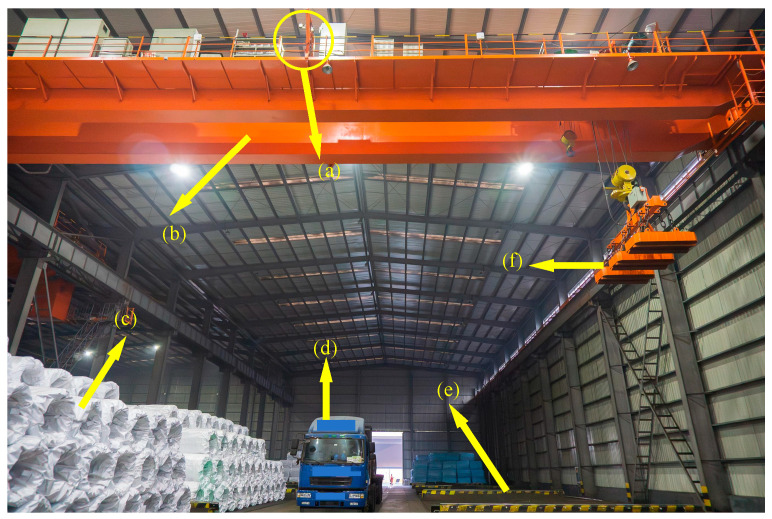
Storage area grabbing equipment diagram (**a**) LIDAR; (**b**) overhead; (**c**) coil stacking; (**d**) truck; (**e**) saddle; and (**f**) crane electromagnet.

The internal setup of the warehouse for storing coil workpieces at the port, including all equipment and the locations for grabbing and placing coils, is depicted in Figure 9. The crane’s grabbing mechanism consists of three electromagnets, which, when electrified, serve as powerful magnets to lift the coils through magnetic attractions manipulated via the cables above. This magnetic grabbing method offers high tolerance for positioning errors, consistent grabbing force, and secure handling compared to mechanical claws or crane hooks. Additionally, a Tianhe 3D laser line scanner is used for LiDAR, positioned in the middle of the crane’s main beam, as shown in Figure 9. Due to the line scanner’s characteristics, the crane must move within a designated target area to accurately capture the complete point cloud data of the stacked coils or trucks, ensuring thorough data acquisition.

Tianhe Electronics’ laser radar product is a high-precision 3D laser scanner designed for large-scale applications such as 3D modeling and the volume measurement of coal piles, stockyards, and traffic monitoring. Unlike traditional line-scanning devices, it uses rotational or solid-state scanning to generate precise 3D point cloud data for large-area, high-accuracy spatial data collection. With a maximum detection range of 200 m, 360° horizontal scanning, and approximately 270° vertical scanning, it is well suited for large environments. The system offers an accuracy of ±2 cm and a 0.06° angular resolution, supporting data sampling at 60,000 points per second. It also features an electric dustproof cover to withstand harsh conditions, ensuring reliable performance even in extreme weather or complex environments.

**Figure 10:**
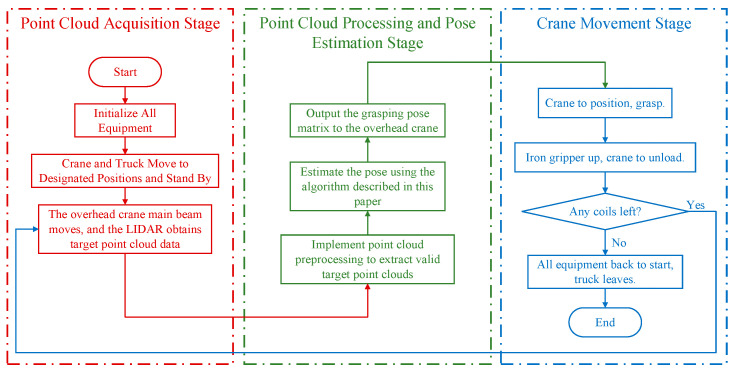
Warehouse overhead crane workflow diagram.

An entire operational workflow in the storage area primarily includes two directions: unloading coils from a fully loaded truck and loading coils onto an empty truck, using the overhead crane to transfer coils between the truck and designated spots on the warehouse floor. The equipment control processes for both directions are similar and include three main stages: (1) the point cloud collection stage, where all equipment is initialized, and the crane’s main beam is moved to align with the line scanner to collect point cloud data; (2) the point cloud processing and pose estimation stage, where point clouds are processed using software and algorithms to extract and handle the target coils for grabbing, and the pose estimation results are sent to the crane; and (3) the crane movement stage, where the crane executes the loading and unloading tasks based on predefined strategies and the pose information, also checking for any remaining coils. This workflow, illustrated in Figure 10, continues in a cycle until all the coils in the target stack are fully loaded or unloaded.

### 4.3. Pose Estimation Result Evaluation

**Figure 11:**
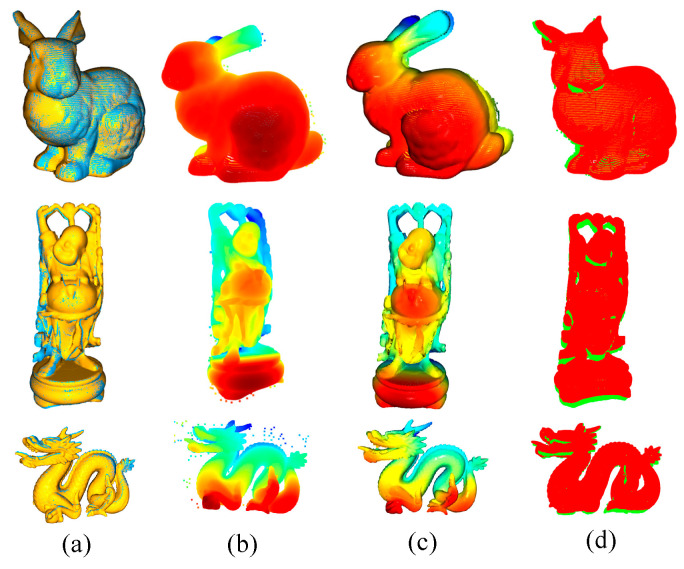
Comparison of the algorithm presented in this paper with other pose estimation algorithms on a public dataset: (**a**) ours; (**b**) Fast-PPF; (**c**) FPFH; and (**d**) PCA + ICP.

When performing pose estimation for coil workpieces, this study first compared the improved algorithm presented in this paper with three commonly used pose estimation algorithms using a public dataset. The algorithms compared included PCA (Principal Component Analysis) [42] coarse registration combined with ICP (Iterative Closest Point) fine registration from the Open3D point cloud processing library, the FPFH (Fast Point Feature Histogram) [21] algorithm, and the Fast-PPF [39] algorithm. The comparisons utilized several datasets from the Stanford 3D Scanning Repository [43]. The experiments with our algorithm and the comparative algorithms are illustrated in Figure 11.

These models were created using the high-precision Cyberware 3030 MS [44] scanner. The figure below demonstrates the experimental results of the algorithm presented in this paper, along with the two comparison algorithms mentioned previously applied to several datasets from the collection. The average errors after registration and the runtime for each algorithm are also displayed in Table 2 below.

Based on the average error and total time metrics from the public dataset using the algorithm presented in this paper and the comparison algorithms, it is evident that all four algorithms achieve pose estimation accuracy at the level of 0.1 mm. This high precision is likely due to the relatively small physical size of the actual objects in the public dataset and the high accuracy of the point clouds obtained. Notably, the algorithm from this paper performed the best with the rabbit dataset, achieving an error of 0.13 mm. The registration results for other point cloud data are also moderately good, with registration times ranging from 15 to 20 s. Although the FPFH algorithm provides sufficient accuracy, such as 0.06 mm for the Buddha model, it also takes the longest time on average, taking 23.68 s for the rabbit model and 21.67 s for the dragon model in particular, which are the longest durations among the four algorithms. On the other hand, the PCA + ICP and Fast-PPF algorithms generally show lower registration precision, with two out of the three models showing lower accuracy than the algorithm from this paper and one showing equivalent accuracy.

In addition to using average distance and time, this paper also includes a comparison based on the more commonly used metrics of recall and accuracy. The Fast-PPF algorithm slightly outperforms the other two methods, especially on the Bunny model, where it achieves 90.5%. However, the proposed method performs excellently in terms of recall, slightly outperforming Fast-PPF on the Bunny model and showing similar results for the other models. In terms of accuracy, although the FPFH algorithm performs well on all three models, the proposed method achieves better accuracy on the Bunny model, reaching 0.05 mm, with accuracy on the other models comparable to that of FPFH.

Therefore, the algorithm from this paper not only shows superior accuracy but also achieves a reasonable balance in terms of time spent, making it an efficient choice among the evaluated methods.

**Figure 12:**
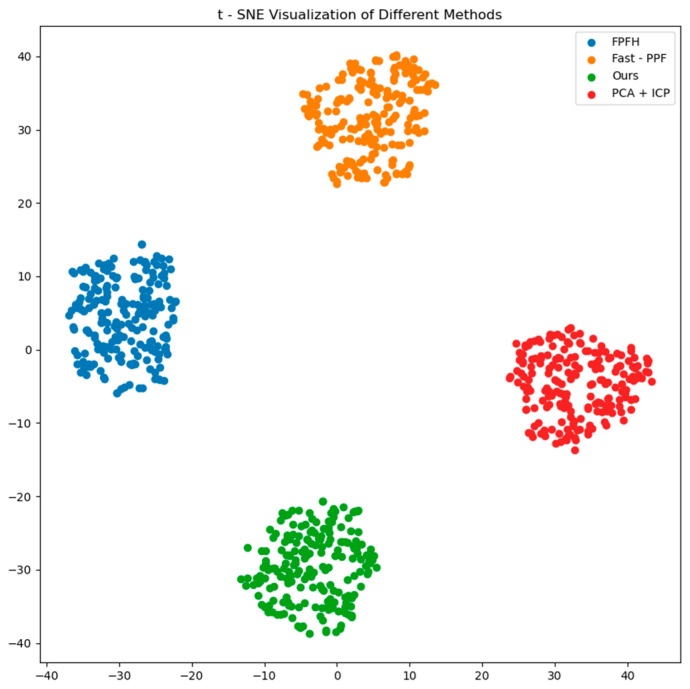
t-SNE visualization demonstrating feature distinction across methods.

Figure 12 presents a t-SNE-based dimensionality reduction visualization, comparing the feature representation capabilities of four methods: FPFH, Fast-PPF, our algorithm, and PCA + ICP. t-SNE maps high-dimensional features into a two-dimensional space while preserving local neighborhood relationships. In the figure, each method forms a distinct and tightly clustered group, indicating that the extracted features have strong discriminative properties.

Notably, the green cluster (representing “Ours”) is clearly separated from the others, highlighting the fact that the features extracted by the proposed method differ significantly from those of the other methods. This difference could be attributed to the unique design of the method in capturing critical features or handling complex data structures. Additionally, the compactness of the green cluster demonstrates the method’s strength in maintaining feature consistency. Overall, this visualization effectively illustrates the potential improvements of the proposed method in feature extraction and emphasizes its uniqueness and effectiveness in high-dimensional feature representation.

**Figure 13:**
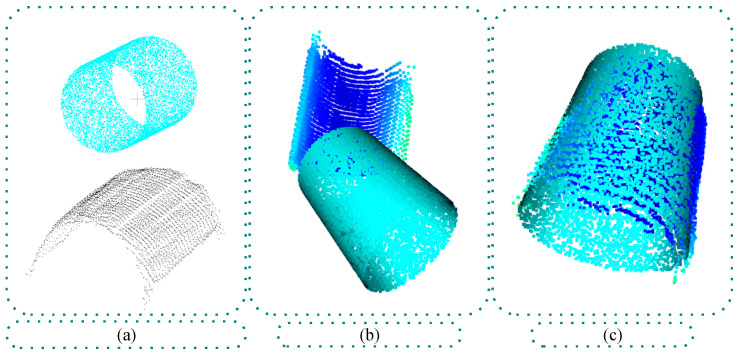
Storage area coil: (**a**) model and scene point cloud diagram; (**b**) coarse registration diagram; and (**c**) fine registration diagram.

After comparing algorithms using the public dataset, the registration results of the algorithm presented in this paper were directly observed through visualized point cloud registration. Additionally, the average error metrics mentioned in Section 3 were used to quantitatively analyze this algorithm to assess its suitability for actual industrial production. The pose estimation using these algorithms was performed on an upper-level machine running Python (version 3.11) under the Windows 11 system, equipped with an Intel Core i7-12700H CPU (Intel, Santa Clara, CA, USA), an NVIDIA RTX 3060 GPU (NVIDIA, Santa Clara, CA, USA), and 16 GB of RAM (Lenovo, Beijing, China). Figure 13 shows the pose estimation results using the algorithm from this paper with a single coil as an example. From left to right, the sequence displays the individual model, the scene point cloud, and the point clouds after coarse and fine registrations. It can be seen that there are some angular and positional discrepancies between the model and scene point clouds during coarse registration. After fine registration, the cylindrical surface of the model point cloud aligns closely with the scene point cloud. The pose matrix output at this point represents the final pose of the scene point cloud.

**Figure 14:**
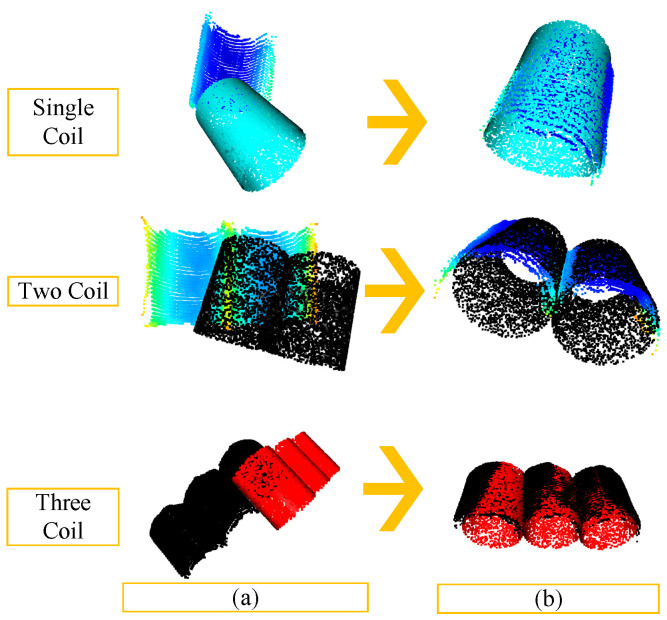
Three quantities of coils corresponding to the registration effect diagram: (**a**) coarse registration and (**b**) fine registration.

Following the scenarios described, pose estimation for two and three coils was performed and compared with the single coil results using the algorithm shown in Figure 13. The results for the coarse and fine registration of one, two, and three coils using this algorithm are displayed in Figure 14, and the average errors post-registration are presented in Table 3 according to the scoring mechanism discussed earlier. Due to the characteristics of the PPF algorithm, the visual quality of registration slightly declined as the number of coils increased, but the overall registration remained satisfactory, with the scene and model point clouds appearing well aligned. Considering the large coil point clouds and the vast space in the storage area, where coils appear neatly stacked without apparent rotational components, the inevitable slight rotation during handling by the overhead crane’s electromagnet and beam system was factored in. These results were taken for a detectable rotation angle component, ensuring a more accurate calculation of the pose matrix for the coil workpieces in the storage area.

Finally, to more clearly illustrate the differences between the algorithm presented in this paper and the two comparison algorithms, the average error introduced earlier was used for quantification. A lower average error indicated higher accuracy during registration, which in turn signified greater precision in pose estimation. Table 3 displays the average errors of pose estimation and the total time required for pose estimation for the algorithm from this paper compared to the other two algorithms.

From the experimental results and accompanying data, it is evident that the algorithm from this paper yielded an average error of 18.5 mm for both one and two coils, constituting only 1.1% of the coils’ total size, and 19.6 mm for three coils, making up 1.2% of the total. These errors are minimal and acceptable for large coil workpieces handled by cranes using electromagnetic technology, ensuring that the grabber can accurately engage and secure the coils for the entire lifting process. The algorithm’s runtimes were 3.6 s for a single coil, 3.4 s for two coils, and 4.7 s for three coils. Similar durations for one and two coils likely stem from the simpler models, which facilitate faster registration or feature finding, while the longer time for three coils is due to additional complexities like multiple touching surfaces and gaps that create more interference in feature matching. However, considering the overall slow operation of cranes from point cloud acquisition to pose estimation and actual grabbing, these times are sufficient to maintain the operational pace required for efficient lifting tasks.

## 5. Summary and Outlook

The improved PPF algorithm proposed in this paper is proposed with the aim of calculating the pose estimation of coil workpieces in actual port warehouses, addressing the challenges posed by the large volume of coils, the abundance of point cloud data, and the slight differences in shape and placement position from typical cylindrical workpieces. The method involves pose estimation and includes both translational and rotational components for positioning. After preparing the point cloud data for the coils, this paper addressed the inadequacies of traditional PPF methods in estimating the pose of large workpieces by proposing an enhanced PPF approach that combines the PPF method with RANSAC for coarse registration and GICP for fine registration, improving the positioning performance of coil workpieces in the storage area. The algorithm was then compared with several common pose estimation algorithms on a public dataset to verify its adequacy and applicability. Finally, the algorithm was applied to actual coil workpiece data to achieve average error proportions of 1.1%—1.2% for single, two, and three coil group poses with algorithm runtimes of 3.6 s, 3.4 s, and 4.7 s, respectively. The results demonstrate good accuracy and robustness, meeting the operational pace required for the automated overhead crane handling of coil workpieces. Future work could involve incorporating deep learning techniques to increase the precision of pose estimation further and enhance the algorithm’s practicality.

## Figures and Tables

**Table 1:** Dimensional data of coil workpieces.

Workpiece Type	Dimension Type	Value (mm)
Coil	Diameter	1200
	Height	1600

**Table 2:** Comparison of algorithms on a public dataset.

Algorithm	Average Error (mm)			Recall(%)			Accuracy(mm)			Total Duration		
	Bunny	Buddha	Dragon	Bunny	Buddha	Dragon	Bunny	Buddha	Dragon	Bunny	Buddha	Dragon
Ours	0.13	0.12	0.12	90.6	89.3	89.0	0.05	0.09	0.10	16.84	21.45	15.49
Fast-PPF	0.14	0.13	0.13	90.5	89.8	90.3	0.11	0.15	0.14	12.61	15.51	17.51
FPFH	0.13	0.06	0.12	87.0	86.2	83.5	0.06	0.08	0.10	23.68	21.67	18.76
PCA + ICP	0.13	0.14	0.12	84.7	85.3	84.1	0.12	0.14	0.13	19.3	15.39	14.29

**Table 3:** Table of pose estimation average errors for the algorithm presented in this paper.

	Average Error (mm)			Time(s)			
	Single	Two	Three	Single	Two	Three	Average Time
Ours	18.5	18.5	19.6	3.6	3.4	4.7	3.9
Proportion	1.1%	1.1%	1.2%				

## Data Availability

Data are contained within the article.
